# The influence of general practitioners on access points to health care in a system without gatekeeping: a cross-sectional study in the context of the QUALICOPC project in Austria

**DOI:** 10.3325/cmj.2019.60.316

**Published:** 2019-08

**Authors:** Kathryn Hoffmann, Aaron George, Tessa Van Loenen, Jan De Maeseneer, Manfred Maier

**Affiliations:** 1 Department of General Practice and Family Medicine, Medical University of Vienna, Vienna, Austria; 2 Department of Community and Family Medicine, Duke Medical Center, Durham, NC, United States; 3 Department of Primary and Community Medicine, IMPULS – Netherlands Center for Social Care Research, Nijmegen, the Netherlands; 4 Department of Family Medicine and Primary Care, Ghent University, Ghent, Belgium

## Abstract

**Aim:**

To assess the rates of specialist visits and visits to hospital emergency departments (ED) among patients in Austria with and without concurrent general practitioner (GP) consultation and among patients with and without chronic disease.

**Methods:**

The cross-sectional questionnaire study was conducted in the context of the QUALICOPC project in 2012. Fieldworkers recruited 1596 consecutive patients in 184 GP offices across Austria. The 41-question survey addressed patients’ experiences with regard to access to, coordination, and continuity of primary care, as well demographics and health status. Descriptive statistics as well as univariate and multivariate regression models were applied.

**Results:**

More than 90% of patients identified a GP as a primary source of care. Among all patients, 85.5% reported having visited a specialist and 26.4% the ED at least once in the previous year. Having a usual GP did not change the rate of specialist visits. Additionally, patients with chronic disease had a higher likelihood of presenting to the ED despite having a GP as a usual source of care.

**Conclusion:**

Visiting specialists in Austria is quite common, and the simple presence of a GP as a usual source of care is insufficient to regulate pathways within the health care system. This can be particularly difficult for chronic care patients who often require care at different levels of the system and show higher frequency of ED presentations.

There is considerable discussion on the impact of regulating access to care as well as appropriate pathways for patients through health care systems. More recently, the value of primary care (PC) as a first point of access has been emphasized, with evidence mounting on the benefit to patients and payers in systems that focus on financial, geographic, and barrier-free access (1-5). Recent research suggests increased benefit in PC sectors that are able to coordinate and guide patients, as opposed to those that allow self-referral by the patient (1-3,6-11). One prominent example is the principle of gatekeeping, in which the access to secondary and tertiary levels of care is only possible through referral from PC. Gatekeeping can contribute to quality control by preventing unnecessary or repeated interventions. Further, it is argued that secondary care is used more efficiently, as primary care providers possess more specific knowledge than patients about the care needed (1,12-14). As patients typically have a limited appreciation of their diagnosis and treatment options, access to secondary care through self-referral can be subject to substantial error (12). Patients with chronic conditions or multiple morbidities, especially older patients, may become lost in the complex pathways of the health care system, particularly in the absence of coordinated referral (15-17). A recent report suggests that even in gatekeeping systems, patients with chronic conditions who could otherwise have been managed by primary care, yet presented for emergency care, cost the National Health Service approximately Ł1.42 billion annually (18). Up to 8–18% of these annual costs could be reduced through investment in primary and community-based services (18). Recent literature further indicates that strongly developed primary health care systems reduce morbidity (7-9). Nevertheless, gatekeeping systems have been criticized for long waiting lists for secondary care services and result in delays in care for conditions such as cancer (19,20). Austria lacks gatekeeping and offers unlimited access to the health care system. Patients can directly access nearly any inpatient or outpatient specialist with no personal financial accountability (21-23). Thus, the situation in Austria is ideal to study the diverse consequences of unregulated access to health care.

In Austria, until 2019 GPs had to be self-employed and work primarily in individual practices together with (health) secretaries. GPs in Austria may work as private doctors as well as have contracts with social health insurance companies. The number of GPs with contracts per region is negotiated between the Austrian Chamber of Physicians and the Main Association of Austrian Social Security Institutions (21). GPs with contracts have a high workload as well as high stress since remuneration is to a great deal due to a fee-for-service scheme. One recent study showed that rural GPs reported an average of 49.3 working hours per week, in addition to 23.7 on-call duties per 3 months and 26.2 out-of-office care services per week (24).

Until 2015, postgraduate GP education could be completed entirely within the hospital. However, since 2015, the last six months of the education occurs in a general practice setting (25). Despite lacking medical specialty status, GPs have been informally recognized as the expected first point of care in the Austrian health care system. While they have the ability to refer patients to specialists, any patient may also choose to directly access any specialty care independent of GP oversight. Unfortunately, GPs are typically only made aware of the clinical information from a specialty visit if the patient decides to share it.

Against this background, the main aim of the present study was to evaluate the rates of patient presentation for specialist visits and visits to hospital emergency departments (EDs) among patients in Austria who do and who do not have a GP. Further, the study assessed these rates in patients with and without chronic disease. A secondary aim was to assess associations regarding the main aim and patients´ demographics to account for possible confounders. The main hypothesis was that having a GP as a usual source of care would lead to lower specialist and ED visits for patients.

## Participants, materials, and methods

### Design

This cross-sectional analysis was conducted in the context of the QUALICOPC study, which as a questionnaire study aimed to assess the quality and costs of primary care in 34 countries in Europe and around the world (26-28). In Austria, data collection took place between November 2011 and May 2012. The target for Austria, established by the QUALICOPC design, was to recruit 180 GPs from all nine federal counties, with representation of sexes, various age groups, and both GPs with and without contracts with public social health insurance companies. The inclusion criterion was that the GP had to have a medical office in Austria; only one GP per office was included. The recruitment process of the GPs in Austria is described in depth elsewhere (28,29). In each of these GP offices, fieldworkers (medical students) attempted to recruit 10 consecutive patients to complete the QUALICOPC patient questionnaires. Relevant for the accuracy of this analysis, nine patients had to complete the patient experience questionnaire and one had to complete the patient value questionnaire. For the present study we used the patient experience questionnaires.

### Questionnaire

The detailed development process of the questionnaire was described by Schäfer et al and was previously tested in a pilot study (27). The final version of the patient experience questionnaire contained 41 questions addressing patients’ experiences with regard to access to, coordination, and continuity of primary care, as well as questions on patient demographics and health status.

The first question used for this analysis was “Do you have your own GP whom you normally consult first with a health problem?” with the answer categories including “Yes, the doctor I just visited,” “Yes, but another doctor in this practice or center,” “Yes, but another doctor from somewhere else,” and “No, I do not have my own doctor.” Later, this variable was dichotomized into the “yes” and “no” answer categories. The second question used was “In the last 6 months, how often have you visited or consulted a GP (this GP or another one)?” with the answer options “This was the first time in the past 6 months,” “Once before this visit,” “2 to 4 times before this,” “5 times or more before this,” and “Don't know.” To evaluate the utilization of specialists, two questions were asked: “How many times in the past 12 months, have you consulted a medical specialist for yourself?” (with the answer categories “Once or twice,” “3 to 5 times,” “6 to 10 times,” and “More than 10 times”) and “After treatment by a medical specialist, my GP knows the result” (with the answer options “Yes,” “No,” and “Don’t know”). Finally, the questions regarding visits to hospital ED were “In the last 12 months, how often did you visit a hospital emergency department for yourself?” with the answer categories “never,” “1 time,” “2 or 3 times,” and “4 or more times” and “Why did you go to the emergency department instead of going to a GP?” with the answer options “I had something GPs do not treat,” “There was no GP available,” “For financial reasons,” “At the emergency department, I expected a shorter waiting time,” “The emergency department provides better care,” “The emergency department is more convenient to reach,” and “Other reason.”

Demographics were assessed in terms of sex, age, highest level of education (none or primary, secondary, tertiary), and country of origin (Austria, another EU 27 country, European country outside the EU, North America/Australia/New Zealand, another country). The country of origin variable was clustered into three groups: Austria, another EU 27 country, and other country. The presence of one or more chronic diseases was assessed with the question “Do you have a longstanding disease or condition such as high blood pressure, diabetes, depression, asthma or another longstanding condition?” with the answer categories “yes” or “no.” All participants were informed about the study procedure and completed a written consent form before participation. The QUALICOPC study was approved by the ethics committee of the Medical University in Vienna (EC N° 808/2011).

### Statistical analysis

Data are presented as absolute and relative numbers. Differences between subgroups were assessed with the χ^2^ test. For the purpose of logistic regression models, self-referral to specialist and hospital ED were dichotomized (having visited a specialist/ED in the past 12 months “yes” or “no”) and defined as dependent variables. Having a usual GP and having a chronic condition were defined as independent variables. All other factors were defined as explanatory variables. First, univariate logistic regression models were conducted consecutively for both dependent and independent variables. Following this, multivariable logistic regression models were run by including all independent and explanatory variables simultaneously. To adjust for a possible inter-practice clustering effect, dummy variables for all GP practice codes were built and included into the models as well. The results of all regression models are presented as odds ratios with 95% confidence intervals. Nagelkerke R˛ (logistic regression models) is presented as a measure of model-fit. Significance level was set at *P* < 0.05. The analysis was performed by IBM SPSS, version 22.0 (IBM Corp., Armonk, NY, USA).

## Results

### Patients

Patients from 184 GP offices were recruited. In summary, fieldworkers contacted 2681 patients (range of 3-30 patients per office) and obtained 1790 completed questionnaires (range of 3-10 per office) with a return rate of 66.8%, 1596 of which were patient experience questionnaires. Table 1 shows the distribution of chronic disease status and patients’ demographics within the sample. The answer to the question “Do you have your own GP whom you normally consult first with a health problem?” in 84.1% (n = 1342) of participants was “yes, the doctor I just visited”, in 2.1% (n = 33) “yes, but another doctor in this practice or center”, in 6.4% (n = 102) “yes, but another doctor from somewhere else”, and in 5.8% (n = 92) it was “no, I don´t have my own doctor”. A total of 1.7% (n = 27) of participants did not answer this initial question and were excluded from further analysis.

**Table 1:** Participants’ demographic characteristics

Variable	Subvariable	n (%)
All		1596 (100)
Chronic condition	yes	639 (40.6)
	no	935 (59.4)
Sex	female	932 (60.4)
	male	612 (39.6)
Age groups (years)	18-40	476 (31.5)
	41-64	663 (43.9)
	65+	372 (24.6)
Highest level of education	no or primary	293 (19.7)
	secondary	966 (64.8)
	tertiary	231 (15.5)
Country of origin	Austria	1330 (86.6)
	another EU 27 country	109 (7.1)
	another country	96 (6.3)

### High number of self-referrals to specialists and emergency departments

More than 90% of patients indicated they did have a usual GP whom they normally consulted first and 85.5% of all participants had visited a specialist at least once in the preceding 12 months (Table 2). In terms of more frequent specialist visits, 38.3% of participants indicated that they had visited a specialist at least three times, and 26.2% had consulted a hospital ED at least once in the previous 12 months. The main reason (37%) identified for directly accessing the ED was the belief that they had a condition that was not treatable by a GP (Table 2). More than one quarter of participants did not believe that GPs were aware of the results of treatments by specialists.

**Table 2:** Participants’ health care utilization*

Variable	Subvariable	All, n (%)	Chronic disease		
			yes, n (%)	no, n (%)	*P*
Do you have your own GP?	yes	1477 (94.1)	605 (95.9)	860 (93.0)	0.020
	no	92 (5.9)	26 (4.1)	65 (7.0)	
In the past 6 months how often have you visited a GP?	0-1	523 (33.7)	120 (19.3)	398 (43.5)	<0.001
	2-4	612 (39.5)	245 (39.5)	362 (39.6)	
	5+	416 (26.8)	256 (41.2)	155 (16.9)	
In the past 12 months how often have you visited a specialist for yourself?	0	232 (14.5)	80 (12.5)	151 (16.1)	<0.001
	1-2	753 (47.2)	243 (38.0)	499 (53.4)	
	3-5	386 (24.2)	196 (30.7)	186 (19.9)	
	6 and more	225 (14.1)	120 (18.8)	99 (10.6)	
After treatment by a specialist did your GP know the results?	yes	1087 (73.1)	457 (78.0)	616 (69.7)	<0.001
	no/don’t know	400 (26.9)	129 (22.0)	268 (30.3)	
In past 12 months how often have you visited a hospital ED for yourself?	0	1136 (73.8)	424 (69.1)	701 (77.1)	0.001
	1-3	391 (25.4)	182 (29.6)	203 (22.3)	
	4 and more	13 (0.8)	8 (1.3)	5 (0.6)	
Why did you go to the ED instead of GP?	Had something GPs do not treat (yes)	142 (37.1)	72 (39.6)	70 (34.8)	0.343
	No GP available (yes)	72 (18.8)	36 (19.8)	36 (17.9)	0.695
	Financial reasons (yes)	0	0	0	-
	Expected shorter waiting time (yes)	7 (1.8)	4 (2.2)	3 (1.5)	0.713
	ED provides better care (yes)	35 (9.1)	23 (12.6)	12 (6.0)	0.032
	ED is more convenient to reach (yes)	41 (10.7)	21 (11.5)	20 (10.0)	0.624
	Other reason(s) (yes)	135 (35.2)	57 (31.3)	78 (38.8)	0.135

*GP – general practitioner; ED – emergency department.

### Chronic disease and self-referrals

Patients with one or more chronic diseases were significantly more likely to have a usual GP and they consulted that GP more frequently (Table 2). Further, patients with a chronic disease had a higher rate of visits to both specialists and the ED than did those without chronic disease. Patients with a chronic disease were more likely to present to the ED, most often because they believed that the ED provided better care.

### Having a usual GP did not decrease the likelihood of self-referrals

In the univariate regression model (Table 3), having a usual GP was not significantly associated with the probability of visits to a specialist, but did demonstrate a significantly increased likelihood of visiting an ED. This association remained consistent when analyzed in the fully adjusted regression model (Table 4).

**Table 3:** Univariate model regarding the self-referral variable*

Variable	Subvariable	Self-referral to specialist		Self-referral to ED	
		OR (95% CI)	*P*	OR (95% CI)	*P*
Do you have your own GP?	yes	1.05 (0.59-1.90)	0.862	2.09 (1.15-3.81)	0.016
	no	1.0		1.0	
Do you have a chronic condition?	yes	1.35 (1.01-1.80)	0.046	1.48 (1.17-1.87)	0.001
	no	1.0		1.0	

*OD – odds ratio; CI– confidence interval; GP – general practitioner; ED – emergency department.

**Table 4:** Adjusted multivariable model regarding the self-referral variable*†

Variable	Subvariable	Self-referral specialist		Self-referral ED	
		OR (95% CI)	*P*	OR (95% CI)	*P*
Do you have your own GP?	yes	1.10 (0.50-2.42)	0.818	2.33 (1.14-4.77)	0.021
	no	1.0		1.0	
Do you have a chronic condition?	yes	1.07 (0.71-1.61)	0.737	1.85 (1.31-2.60)	<0.001
	no	1.0		1.0	
Sex	female	1.44 (1.01-2.05)	0.045	0.90 (0.66-1.22)	0.484
	male	1.0		1.0	
Age groups	18-40	0.99 (0.58-1.70)	0.971	3.13 (2.00-4.90)	<0.001
	41-64	1.04 (0.64-1.68)	0.887	1.08 (0.73-1.60)	0.714
	65 and older	1.0		1.0	
Highest level of education	primary	1.0		1.0	
	secondary	1.98 (1.27-3.10)	0.003	1.08 (0.72-1.62)	0.705
	tertiary	1.69 (0.92-3.11)	0.089	0.74 (0.43-1.28)	0.285
Country of birth	Austria	1.0		1.0	
	another EU 27 country	1.29 (0.65-2.59)	0.470	1.18 (0.67-2.08)	0.575
	others	1.07 (0.46-2.45)	0.881	1.32 (0.70-2.51)	0.397
In the past 12 months how often did you visit a GP?	0-1	1.0		1.0	
	2-4	1.99 (1.33-2.96)	0.001	1.33 (0.92-1.92)	0.127
	5 and more	3.09 (1.88-5.08)	<0.001	2.53 (1.68-3.82)	<0.001
Nagelkerke R^2^		0.304		0.291	

*OD – odds ratio; CI – confidence interval; GP – general practitioner; ED – emergency department.

†Adjusted for GP practice code.

### Having a chronic condition increased the probability of self-referrals

Patients with a chronic disease demonstrated a significantly higher likelihood of visiting specialists and an ED in the univariate regression model (Table 3). However, in the fully adjusted regression model, having a chronic disease failed to show a significant association with specialist visits (Table 4).

Other factors significantly and positively associated with specialist visits were female sex, a secondary educational level, and three or more visits to a GP in the previous 12 months (Table 4). Factors positively associated with visits to the ED were the age between 18 and 40 years and visiting a GP five or more times in the previous 12 months (Table 4).

## Discussion

This study demonstrates a high rate of specialist visits among both patients with and without chronic disease. This was identified even in the context of more than 90% of patients having a usual GP whom they normally consult first with a health problem. Such high utilization rates of specialists in the Austrian health care system by the general population have been identified previously, with rates between 63% and 67.4% (22,23,30). Compared with countries with gatekeeping systems, Austria demonstrates a two- to 3-fold higher specialist-consulting rate (23,31). Such a high utilization rate of specialists may be explained by the phenomenon of a supply-induced market, particularly in a country without regulated pathways between primary and secondary health care and with no financial cost of care to patients. Effectively, patients will tend to use more specialists when they are easier to access (22). Thus, for example, such patients will go directly to a gynecologist for a simple PAP-smear or to an orthopedist for chronic low back muscle spasm (23,30). Consequently, GPs perform fewer primary clinical care activities, such a simple gynecological check-ups because nearly all women visit gynecologists directly (30). This results is an inverse shift in the spectrum of care and – as opposed to physicians working to the highest level of their specialty, skills, and license, many are burdened with a significant percentage of primary and more mundane clinical tasks. This opposes the general goal in health care delivery to have every team member working to the top of his or her competence and skillset, and to optimize access such that the right patient is seen by the right provider at the right time.

Another result found in this study is that a quarter of patients surveyed did not believe that their GP was aware of the results from visits to specialists. This is a fundamental flaw in unregulated access to care in Austria and elsewhere, as GPs usually will only receive results from specialists if the referral was issued by the GP. For astonishing 85% of patients that choose to seek first consultation with a specialist, the GP will not be formally informed of findings, results, or medications prescribed (21). This represents a particular challenge for patients with chronic diseases who utilize specialists directly with higher frequency. These patients are in danger of both getting lost in a complex system and losing continuity of care through regular communication with their GP, and are also at increased risk for duplicative diagnostic testing and polypharmacy (15,32).

Additionally, presentation to the ED was found to be quite high (26.2% of all participants) and even higher in patients with chronic diseases. Van Loenen et al (33) found that the number of hospital beds had a greater impact on avoidable hospitalizations than effective primary care, and in fact, Austria has one of the highest hospital admission rates in the European Union (34-36). Along these lines, patients in this study suggested that the most common reason why they presented directly to the ED was “because GPs cannot treat this.” While this could represent a rational reason for visiting the ED, perhaps this does not match up to reality, and patients might be unaware of the spectrum of care adequately provided by GPs. One recent study in Austria revealed that only 20%-50% of patients believed they could visit a GP for treatment for psychological conditions, social problems, visual impairment, or even wound treatment, such as suturing a wound or wart removal (37). Further, nearly one in five patients presented to the ED because there was no GP available, and one in ten because the ED was more convenient. These results suggest that there remains room for improvement with regard to increasing open hours for care and expanding access to primary care in Austria. However, 12.6% of patients with chronic diseases believed that EDs provide better care, and more than a third of participants marked “another reason.” These two answers suggest that further analysis of this problem would be beneficial.

Our findings are consistent with those from other countries with rather weak primary health care systems in terms of coordination and continuity of primary care, such as Germany (31). These countries might endanger the health of their population, as evidence suggests that strongly developed PHC systems are associated with better population health, lower rates of unnecessary hospitalizations, and relatively lower socioeconomic inequality (7,38). Austria and similar countries also espouse relatively high costs for the health care sector and maintain among the highest hospital admission rates in the European Union (34).

An outstanding result of this study is the lack of an inverse association between patients with a usual GP whom they normally consult first and the likelihood of specialist visits. Further, and perhaps more concerning, these patients had an increased likelihood of visits to the ED. However, results were similar for patients with chronic disease, who demonstrated an increased likelihood of visits to the ED. Therefore, the sheer presence of a usual GP, which is politically and informally promoted as the first point of contact and coordinator of care, is insufficient to regulate pathways of patients within the health care system. This is particularly evident for patients with chronic conditions, who otherwise would benefit most from appropriate pathways of care (7,15,32,39).

Our findings have implications for health care system planning, for reduction of unnecessary utilization, as well as optimization of workforce including the training of future GPs and the reduction of global costs. In essence, for the primary care sector to be effective in providing both coordinated and continuous care there must either be incentives for patients to follow optimal pathways through the system or barriers to prevent inappropriate access.

At the provider level, better-organized and sufficiently funded primary care teams, as well as extended and more patient-centered office hours, could improve care, patient recognition of GPs´ scope of practice, and ultimately referral rate. One nationwide UK study suggested that smaller practice size is associated with increased likelihood of presentation to the ED (40), whereas practices with same day turnaround of laboratory tests were associated with a reduced ED attendance (41). Beyond organizational and systemic changes, providers and primary care teams will require state-of-the-art education and training in order to take on the role as advanced care coordinators or gatekeepers. Lastly, system level changes should be implemented, including the establishment of a gatekeeping process, list system, and/or financial incentives.

The study sample consists of participants recruited in a GP office on a voluntary base, which may have led to a selection bias, with the possibility that the study failed to contact or include those patients who avoid or bypass GP care altogether. Therefore, the number of specialist visits may have been underestimated and the percentage of patients with a usual GP may have been overestimated. Contrary, the findings could be overestimated due to the fact that the study participants completed the questionnaire retrospectively, which could have led to a memory bias. Moreover, the reason for specialist visits was not completely assessed, aside from the recognition of presence or absence of chronic disease at the time of the visit. Finally and probably most important, generalization of the results to the Austrian population has to be made with caution as the distribution of the demographics of the patients´ sample is not representative of the Austrian population, but rather represents a close approximation.

In Austria, a country without a gatekeeping system, where direct access to higher levels of care is open and nearly fully covered, patients access secondary care often without medical necessity. This study demonstrated that the simple presence of a GP as a usual source of care for first contact is insufficient to guarantee coordinated, continuous, and integrated care at the primary care level. Health sectors should embrace additional measures to support regulation through the health care system at patient, provider, and systems levels. In order to overcome the documented weakness of the PHC system in Austria and strengthen it, and to reduce costs and avoid unnecessary access to higher levels of care, GPs must be put in a position to effectively guide patients through optimal pathways of care and to coordinate health care for the benefit of all – patients, providers, and payers.
